# Human m^6^A-mRNA and lncRNA epitranscriptomic microarray reveal function of RNA methylation in hemoglobin H-constant spring disease

**DOI:** 10.1038/s41598-021-99867-9

**Published:** 2021-10-14

**Authors:** Heyun Ruan, Fang Yang, Lingjie Deng, Dongmei Yang, Xiaoli Zhang, Xueyu Li, Lihong Pang

**Affiliations:** 1 Department of Obstetrics and Gynecology, The First Affiliated Hospital of Guangxi Medical University, No. 6 Shuangyong Road, Nanning, Guangxi China; 2 Department of Obstetrics and Gynecology, Minzu Hospital of Guangxi, Zhuang Autonomous Region, Affiliated Minzu Hospital of Guangxi Medical University, Nanning, Guangxi China

**Keywords:** Biochemistry, Biological techniques, Genetics, Molecular biology, Biomarkers, Diseases, Health care

## Abstract

The thalassemia of Hemoglobin H-Constant Spring disease (HbH-CS) is the most common type of Thalassemia in non-transfusion thalassemia. Interestingly, the clinical manifestations of the same genotype of thalassemia can be vastly different, likely due to epigenetic regulation. Here, we used microarray technology to reveal the epigenetic regulation of m^6^A in modifiable diseases and demonstrated a role of ***BCL2A1*** in disease regulation. In this study, we revealed that methylating enzyme writers including ***METTL16***, ***WTAP, CBLL1, RBM15B,*** and ***ZC3H13*** displayed low expression and the demethylating enzyme ***ALKBH5***, along with reader proteins including ***IGF2BP2*** and ***YTHDF3*** exhibited high expression. In addition, ***BCL2A1*** was hypo-methylated and showed low expression. We also revealed that the ***BCL2A1*** methylation level and ***IGF2BP2*** expression were negatively correlated. Additionally, the mRNAs expression between ***ALKBH5*** and ***IGF2BP2*** were positively correlated. In HbH-CS, most genes were hypo-methylated. This included ***BCL2A1***, which may play an important role in the process of red blood cell differentiation and development of HbH-CS. Moreover, the mRNA-M^6^A methylation status may be regulated by the demethylating enzyme ***ALKBH5*** via ***IGF2BP2***.

## Introduction

Thalassemia is a serious genetic hemolytic anemic disease that destroys human health and brings about disability and/or death. Various forms of this disease are caused by a defect in the Globin gene, which reduces or completely ceases globin chain synthesis, thereby creating an imbalance in the chain/non-chain ratio of hemoglobin formation^1^. Thalassemia is one of the most common single gene diseases in the world, accounting for more than 5% of cases worldwide^2^. The severity of this disease is judged by the need for transfusion. Patients with thalamassia, is therefore, grouped into one of two categories: transfusion dependent thalassemia (TDT) and non-transfusion dependent thalassemia (NTDT)^2–5^. NTDT can further be broken into 3 clinically discrete categories: β-thalassemia intermedia, hemoglobin E/β-thalassemia (mild and moderate forms), and α-thalassemia intermedia (otherwise known as, α-thalassemia or hemoglobin H disease)^5^. Annually, ~ 10,000 births carry the α-thalassemia intermedia form of NTDT^6,7^. Among them, the Hemoglobin H Constant Spring (HbH-CS) is the most common non-deletion form of the Haemoglobin H disease. Moreover, it is more severe in nature, as compared to thalassemia alone^2^. In view of the severe clinical phenotype of HbH-CS and its related complications, there is a growing consensus that HbH-CS is a disease with poor prognosis. Similar to the absence of HbH, HbH-CS requires special attention and perfomalized therapy^4^. Unfortunately, historically, thalassemia intermedia, was deemed as a milder form of NTDT. Hence, patients with HbH-CS were administered few to no transfusion and thereby had little to no iron chelation. However, with evidences from multiple long-term clinical studies, it is now clear that NTDT, which do not require blood transfusions early in life, can develop life threatening complications later in life and must, therefore, be monitored and managed with care^8^. The clinical manifestations of HbH-CS thalassemia greatly vary in severity and cannot be explained solely by the causative genes related to thalassemia intermedia^3^. Mild patients have only mild anemia, normal growth and development, and do not require blood transfusion and splenectomy. In contrast, severe patients have moderate to severe anemia, jaundice, hepatosplenomegaly, thalassemia appearance, backward growth and development, low resistance, can be complicated with infection, iron overload, cholelithiasis, folic acid deficiency, fracture, etc. In addition, oxidative drugs and infection can induce hemolytic crisis^9^. Hence, the effect of epigenetic modification on HbH-CS must be considered.

Epigenetics, which includes DNA, RNA, and protein modifications is often used to link genetic modifications to disease phenotypes^10,11^. Recently, DNA methylation was reported to be involved in thalassemia or hematopoietic diseases^12–14^. However, there are no reports on whether RNA methylation plays a role in thalassemia. Nevertheless, RNA methylation has been implicated in other circulatory diseases^15–17^. Hence, in this study, we analyzed the link between epigenetics and the HbH-CS phenotype, using the human m^6^A-mRNA and lncRNA epitranscriptomic microarray data from the immature erythrocytes of 5 HbH-CS thalassemia (T) and 5 normal healthy volunteers (N). Our goal was to elucidate the underlying mechanism behind HbH-CS pathogenesis.

## Results

### M^6^A-mRNA in HbH-CS and healthy volunteers

**Figure 1:**
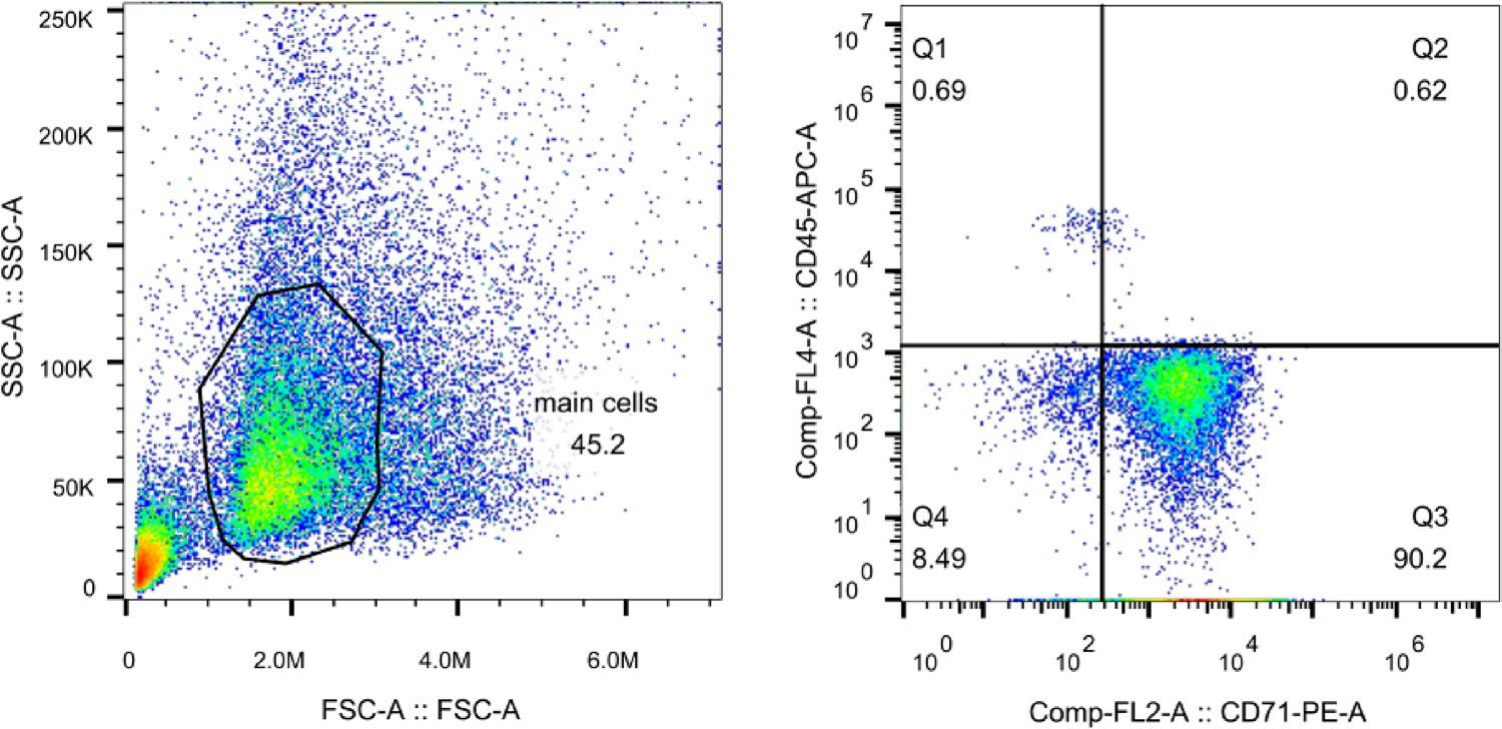
Confirmation of red blood cell sorting, using flow cytometry. Aggregation of signals in the Q3 region is indicative of a mostly (90.2%) CD71+ (denoting RBC) cell population.

**Figure 2:**
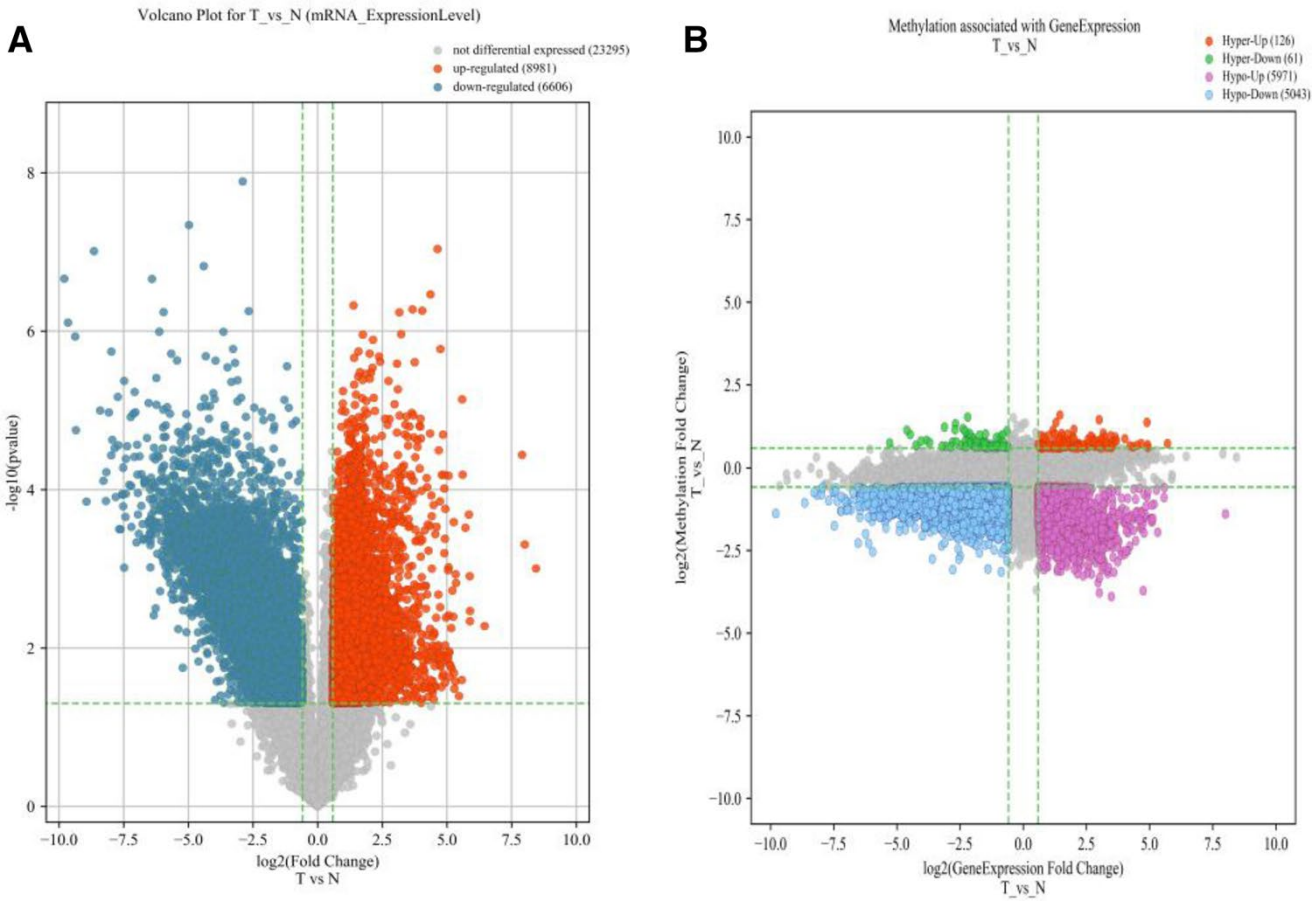
Expression and methylation profiles of mRNAs in immature RBCs of Hb cs thalassemia (T) and healthy volunteers (N).

We observed no discernible differences in the age or gender of the HbH-CS thalassemia (T) and healthy volunteers (N) cohorts (Table 1). Using flow cytometry we determined that our samples were primarily composed of immature RBCs (Fig. 1). Based on our m^6^A-mRNA and lncRNA epitranscriptomic microarray analysis, there were 8981 up-regulated RNA and 6606 RNA that were either down-regulated or showed no differential expression between the T versus N samples (Fig. 2A). Moreover, comparing differential RNA methylation between the two cohorts, we observed 126 RNAs that were heavily methylated, 61 RNAs that had very little methylation, 5971 RNAs that had slight elevation in methylation, and 5043 RNAs that exhibited a slight reduction in methylation (Fig. 2B).

**Table 1:** Clinical information of all participants.

Characteristics		N (n = 16)	T (n = 15)	P
Age (years)		17.8 ± 14.29	22.44 ± 14.16	*P* = 0.372
Sex	Female	7	9	*P* = 0.366
	Male	9	6	

N represents healthy volunteers (n = 16) and T refers to the HbH CS thalassemia patients (n = 15). T-test was used for analysis. No discernible difference was observed in age and gender within the N and T cohorts.

### Differential expression of genes in T versus N

**Figure 3:**
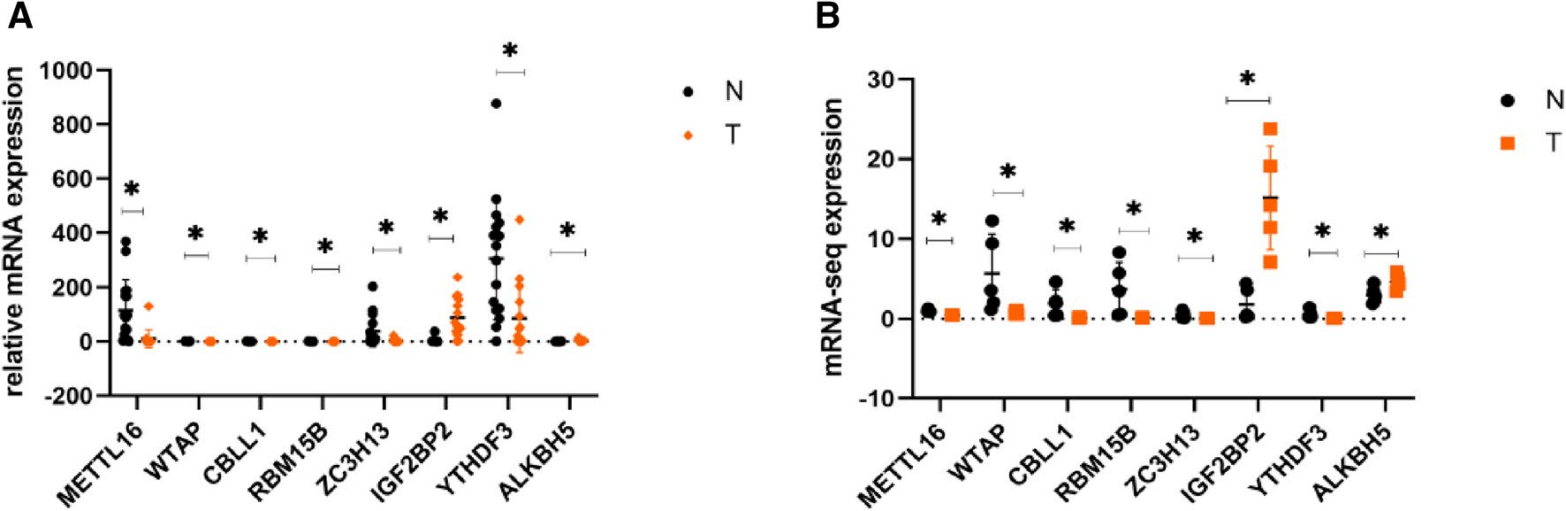
The differentially expressed profile of m6A-mRNAs in immature red blood cells of Hb CS thalassemia (T) and healthy volunteers controls (N) (*P < 0.05.). Relative mRNA expression, as evidenced by qRT-PCR. The qRT-PCR data (**A**) was consistent with the epitranscriptomic rnicroarray sequence (**B**).

Based on our analysis, we selected 8 differentially expressed mRNAs, based on their m^6^A level, and confirmed their expression patterns in T (n = 15) and N (n = 16) samples, using RT-qPCR. The selected mRNAs were ***Mettl16***, ***WTAP, CBLL1, RBM15B, ZCH3H13, IGF2BP2, YTHDF3***, and ***ALKBH5***. As depicted in Fig. 3A, these genes expressed differently in T versus N. However, their expression patterns were consistent with the epitranscriptomic microarray sequences (Fig. 3B), thereby confirming the reliability of the microarray technique. The primers for our RT-qPCR work are presented in Table 2.

**Table 2:** Primer sequences used for RT-PCR.

Gene name	Primer	Sequenceproduct	Size (bp)
METTL16	Forward	5′AGTACCATCACCACCAAGTAAG 3′	161
	Reverse	5′TTTCAATCCATGTCGTGACAAC 3′	
WTAP	Forward	5′CTGACAAACGGACCAAGTAATG 3′	93
	Reverse	5′AAAGTCATCTTCGGTTGTGTTG 3′	
CBLL1	Forward	5′ACAAGCACCATATGAGCCATAT	95
	Reverse	5′TGGCTGATTATAGTGCTCATGT	
RBM15B	Forward	5′ATCTTTCAGAGTACGCTCAGAC	93
	Reverse	5′CTAGGATATGCATAGACGTGGG	
ZC3H13	Forward	5′GATCAGTTAAAGCGTGGAGAAC 3′	177
	Reverse	5′CTCTCTGTCGTGTTCATATCGA 3′	
IGF2BP2	Forward	5′GATGAACAAGCTTTACATCGGG3′	202
	Reverse	5′GATTTTCCCATGCAATTCCACT3′	
YTHDF3	Forward	5′GCTCCACCAACCCAACCAGTTC3′	144
	Reverse	5′CTGAGGTCCTTGTTGCTGCTGTG3′	
ALKBH5	Forward	5′GCAAGGTGAAGAGCGGCATCC3′	128
	Reverse	5′GTCCACCGTGTGCTCGTTGTAC 3′	
β-actin	Forward	5′GTGGCCGAGGACTTTGATTG 3′	73
	Reverse	5′CCTGTAACAACGCATCTCATATT 3′	

### Gene ontology enrichment and pathway analysis

**Figure 4:**
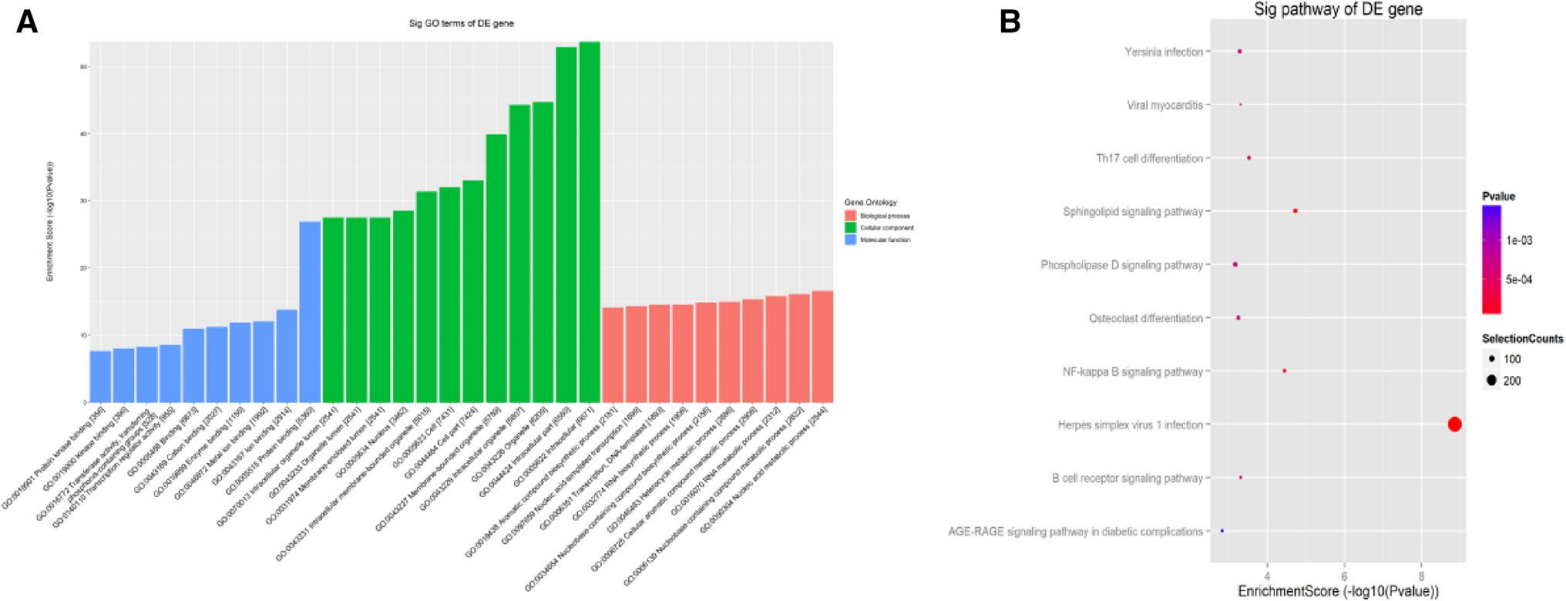
The top ten enrichment scores of significant enrichment hypo-methylated genes ontology (in bar format) depicted genes from the biological process, cell component, and molecular function (**A**). Significance was established at p-value 0.05. The top ten enrichment scores of significant enrichment hypo-methylated genes pathway (− log10 (Pvalue)) (**B**), in dot format. The degree of red corresponds to significance. The size of the dots corresponds to mRNA entities that are directly linked to the noted Pathway IDs.

To explore the functional correlations between the differentially m^6^A-methylated and differentially expressed mRNA, we enriched select gene ontological functions and GO terms (http://www.geneontology.org) and used the bioconductor top GO package for analysis. Among the top 10 GO were biological process (BP), cellular component (CC), and molecular function (MF). Moreover, we determined significance using Fisher Exact test *p*-value and established the enrichment score via -log10 (*p*) formula. Figures 4A illustrates the differentially m^6^A hypo-methylated mRNA. In addition, we conducted metabolic pathway analysis, using KEGG pathways, on the differentially m^6^A hypo-methylated mRNAs, with statistical analysis as described before. As depicted in Fig. 4B, the pathway enrichment analysis showed involvement of essential pathways like the Herpes simplex virus 1 infection, sphingolipid signaling pathway, NF-kappa B axis, Th17 cell differentiation, B cell receptor axis, Viral myocarditis, Yersinia infection, osteoclast differentiation, phospholipase D axis, and the AGE-RAGE axis.

### BCL2A1 expression is regulated by m^6^A modification

The “m^6^A methylation level” for all transcripts was calculated as the percentage of Modified RNA (% Modified) in all RNAs based on the immunoprecipitated (IP) (Cy5-labelled) and supernatant (Sup) (Cy3-labelled) normalized intensities:

% Modified = (modified RNA)/(Total RNA) = IP/ (IP + Sup) = 〖IP〗_(Cy5 normalized intensity)/(〖IP〗_(Cy5 normalized intensity) + 〖Sup〗_(Cy3 normalized intensity) ).

**Figure 5:**
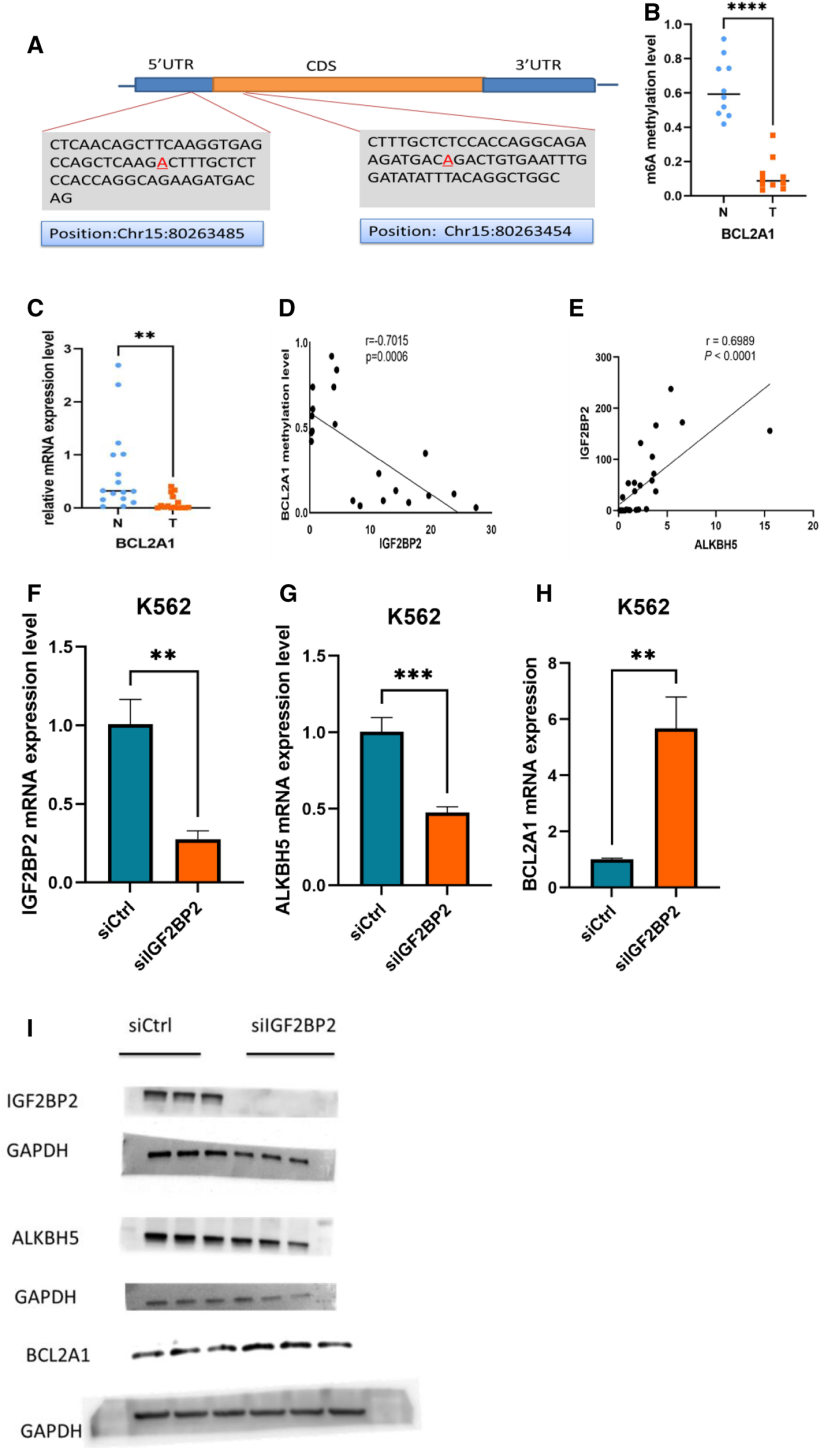
BCL2A1 mRNA levels are modulated by m6A associations. (**A**) An illustration of m6A sites in BCL2A1^21^ (http://m6avar.renlab.org/) (**B**) The m6A-BCL2A1 association was severely hypo-regulated (P < 0.0001). (**C**) BCL2A1 transcription was markedly reduced in T versus N (P = 0.0085). (**D**) BCL2A1 methylation status and IGF2BP2 levels were negatively correlated (r = − 0.7015, p = 0.0006). (**E**) Transcriptional relationship between ALKBH5 and IGF2BP2 (r = 0.6989, p < 0.0001). P-values were derived from Student's t-test and Correlation Analysis. (**F**–**H**) qRT-PCR analysis of IGF2BP2, ALKBH5 and BCL2A1 in K562 transfected with siRNA negative control (siCtrl), IGF2BP2 siRNA. (**I**) western blot analysis of IGF2BP2, ALKBH5 and BCL2A1 in K562 transfected with siRNA negative control (siCtr1), IGF2BP2 siRNA.

The top 20 differentially hypo-methylated m^6^A mRNAs in T versus N are listed in Table 3 and 20 differentially hypo-methylated m^6^A non- mRNAS including lncRNA and other small RNAs are listed in Table 4. Here, we evaluated m^6^A levels of BCL2A between T (n = 10) and N (n = 10). The primary methylated sites were in the CDS and 5’UTR regions^21^. Figure 5A illustrates the methylated mRNA and positions of methylation. Based on our data, we demonstrated that the mRNA expression and m^6^A levels of BCL2A1 were markedly down-regulated in T versus N (Fig. 5B,C). The primers used for BCL2A1 identification were 5′AGAATCTGAAGTCATGCTTGGA3′and 5′CTCCTTTTCCATCACTTGGTTG3. In addition, using KEGG analysis, we showed that the methylation of BCL2A1 was linked to IGF2BP2 mRNA expression (Fig. 5D). Figure 5E showed that ALKBH5 is positively correlated with IGF2BP2. To investigate the the potential roles of IGF2BP2, we examined the effects of IGF2BP2 knockdown in K562 cells and confirmed that IGF2BP2 was knocked down by using siRNA sequences (Fig. 5F,I). We found that knockdown of IGF2BP2 significantly inhibited the ALKBH5 and BCL2A1 expression of k562 cells (Fig. 5G–I).

**Table 3:** The top 20 differentially hypo-methylated m6A-mRNAs in HbH CS thalassemia.

Gene name	Regulation	Foldchange (log2-Scaled) m6A	Foldchange (log2 -Scaled)GE	Locus
BCL2A1	Hypo-down	− 3.1482429	− 0.873422863	chr15:80,253,234–80,263,511:-
CD93	Hypo-down	− 3.0826079	− 2.790594724	chr20:23,059,986–23,066,977:-
ARHGAP4	Hypo-down	− 3.0612382	− 1.397050765	chrX:153,172,831–153,191,698:-
SUSD1	Hypo-down	− 2.8111023	-0.646360165	chr9:114,803,065–114,937,688:-
MYO1F	Hypo-down	− 2.765078	− 2.551049185	chr19:8,585,674–8,642,331:-
SLA	Hypo-down	− 2.7599988	− 3.59682397	chr8:134,049,898–134,115,156:-
TOM1	Hypo-down	− 2.6615412	− 2.495029988	chr22:35,695,797–35,743,987: +
ARSG	Hypo-down	− 2.5774009	− 0.852254218	chr17:66,255,323–66,418,872: +
MEFV	Hypo-down	− 2.574598	− 1.736043206	chr16:3,292,028–3,306,627:-
G0S2	Hypo-down	− 2.5421099	− 5.958020173	chr1:209,848,765–209,849,733: +
RP11-80B17.1	Hypo-up	− 3.8966634	3.489528549	chr3:161,214,596–161,221,730: +
TLR10	Hypo-up	− 3.7814104	3.013192068	chr4:38,773,860–38,784,611:-
TTC30B	Hypo-up	− 3.7168964	4.747257284	chr2:178,413,726–178,417,742:-
PAM	Hypo-up	− 3.4721222	2.95986846	chr5:102,201,714–102,364,814: +
UACA	Hypo-up	− 3.3243618	3.019706944	chr15:70,949,141–70,994,647:-
DDX58	Hypo-up	− 3.2698532	2.377325024	chr9:32,455,300–32,502,734:-
GOLGA1	Hypo-up	− 3.2181953	2.705210092	chr9:127,640,636–127,703,378:-
ADCY3	Hypo-up	− 3.2063213	2.140407984	chr2:25,042,041–25,142,055:-
CHRNA1	Hypo-up	− 3.1912752	3.351292425	chr2:175,612,388–175,629,189:-
LIPF	Hypo-up	− 3.1811642	2.530582545	chr10:90,424,215–90,438,571: +

**Table 4:** The 20 differentially hypo-methylated m6A other RNAS including lncRNA and other small RNAs.

Type	Regulated	Foldchange	Pvalue(unpaired t-test)	GeneSymbol	Locus
lncRNA	hypo	0.666631912	0.001001122	RP4-651E10.4	chr1:87,036,864–87,170,176:-
lncRNA	hypo	0.666561648	0.002866294	RP11-552F3.10	chr17:73,893,141–73,896,229: +
lncRNA	hypo	0.66615389	0.043268439	PRIMPOL	chr4:185,570,767–185,616,113: +
lncRNA	hypo	0.666014095	0.036625819	FAM185A	chr7:102,389,399–102,449,672: +
lncRNA	hypo	0.665494975	0.007337869	RP11-378I13.1	chr1:57,289,352–57,292,593:-
lncRNA	hypo	0.665279091	0.00670786	AMPH	chr7:38,431,589–38,468,885:-
lncRNA	hypo	0.665043357	0.02109388	PTPN21	chr14:88,959,244–89,017,833:-
lncRNA	hypo	0.664971159	0.001343962	CTD-2382E5.1	chr15:42,264,961–42,291,292: +
lncRNA	hypo	0.664702615	0.029267432	RP11-58H15.4	chr4:144,434,625–144,435,788:-
lncRNA	hypo	0.664663218	0.010643021	PRM2	chr16:11,369,493–11,370,337:-
pri-miRNA	hypo	0.666116893	0.016809979	pri-5-hsa-mir-6738	chr1:155,921,117–155,921,217:-
pre-miRNA	hypo	0.665986206	0.045864605	hsa-mir-6820	chr22:38,363,570–38,363,631: +
pri-miRNA	hypo	0.665974655	0.01367578	pri-5-hsa-mir-6857	chrX:53,432,687–53,432,787:-
pre-miRNA	hypo	0.665876082	0.023250338	hsa-mir-130b	chr22:22,007,593–22,007,674: +
pri-miRNA	hypo	0.665669961	0.036412869	pri-3-hsa-mir-5694	chr14:67,908,482–67,908,582:-
pre-miRNA	hypo	0.665343126	0.019019336	hsa-mir-6814	chr21:43,166,932–43,167,001:-
snoRNA	hypo	0.663864549	0.003881281	RF00322	chr14:42,063,666–42,063,794: +
pri-miRNA	hypo	0.663166661	0.005558968	pri-3-hsa-mir-4675	chr10:20,840,965–20,841,065: +
pre-miRNA	hypo	0.662849078	0.019664973	hsa-mir-609	chr10:105,978,547–105,978,641:-
pri-miRNA	hypo	0.662250133	0.010265539	pri-3-hsa-mir-4529	chr18:53,146,519–53,146,619: +

## Discussion

In recent years, it became evident that people carrying the same Globin genotype can have vastly different phenotypes. Moreover, thalassemia can aggravate in presence of stressors like pregnancy, infection, surgery and so on. Based on these facts, it is possible that thalassemia may be affected by other, non-linked genes^19^. Multiple reports have confirmed that cis-regulatory elements like DNA methylation, histone acetylation, and micro RNAs can regulate pathogenesis and clinical heterogeneity of thalassemia^19–21^. In addition, other modifiers were also shown to affect thalassemia phenotype, such as mutations in the molecular chaperone haemoglobin stabilizing protein (AHSP) and transcription regulator GATA1. These emerging reports offer novel view into the therapeutics and prevention of thalassemia, including prevention of the disease in thalassemia heterozygote carriers^22^.

Recent studies on m^6^A-mediated RNA modification demonstrated the dynamic reversibility of RNA modification in regulating RNA metabolism, processing, and directional differentiation of stem cells^23^. Moreover, the m^6^A methylation was shown to modulate the coordinated efforts of transcription and post-transcriptional expression^24^. This includes events like mRNA splicing, export, localization, translation, and stability. M^6^A-mediated methylation of mRNA can potentially enable interaction with modulatory proteins which can regulate downstream gene expression. Emerging evidences suggest a crucial role of m^6^A-mediated mRNA modification in driving mRNA translation^25^.

To determine whether m^6^A modification is involved in producing the thalassemia phenotype, we assessed immature erythrocytes from the peripheral blood of 15 T and 16 N. We identified that methylating enzymes like ***METTL16***, ***WTAP***, ***CBLL1***, ***RBM15B***, and ***ZC3H13*** were scarcely expressed and the demethylating enzyme ***ALKBH5***, along with reading protein-related genes, such as, ***IGF2BP2*** and ***YTHDF3***, were highly expressed in T versus N. In addition, we detected differential regulation of other m^6^A-methylated RNAs like ***RBM15***, ***YTHDF3***, *I****GF2BP1***, *IGF2BP3*, and multiple mRNAS, micRNA, and LNC RNA, which were hypo-methylated in T versus N. Given these evidences, we believe that the m^6^A-mediated methylation is crucial for the pathogenesis of HbH-CS.

BCL2A1 (B cell lymphoma 2 related A1), from the BCL2 (B cell lymphoma 2) protein family^26^, counters cell apoptosis via prevention of cytoplasmic accumulation of cytochrome and prevents the subsequent stimulation of the internal apoptotic axis. This protein is often highly expressed in advanced cancers and denotes poor prognosis^27^. In this study, we verified that the BCL2A1 mRNA was hypo-m^6^A-methylated and had low expression in T versus N. However, the underlying mechanism is yet to be determined. M^6^A methylation can stabilize target mRNA, thereby affecting downstream gene expression^28,29^. M^6^A methylation is generally common in protein-coding transcripts and are localized in the 3′UTRs^29^ and 5′UTR^30^ regions. Interestingly, the methylation sites of BCL2A1 are located within the CDS^21^ and 5'UTR regions^18^.

Based on our data, we propose that alterations in the mRNA methylation status contributes to thalassemia. Due to the severe down-regulation of BCL2A1 in HbH-CS patients, apoptosis remains uninhibited, leading to hemolytic anemia. In thalassemia, erythropoiesis is finely regulated by a complex network of transcription factors, including those involved in erythropoiesis like the erythropoietin receptor EPOR, glycophorin, and Globin peptide chains. In addition, the anti-apoptotic protein BCL-x, induced by the transcription factors STAT5 and GATA1, are activated by erythropoietin EPO^31^. When BCL2A1 levels diminish during erythroid development, further erythrocytes production ceases, thereby aggravating thalassemia. Based on our analysis, we detected BCL2A1 in 3 separate signaling pathways, namely, NF-KappaB, Acute Myeloid Leukemia and transcriptional misregulation in cancer (Supplemental Information). Interestingly, we also discovered that the m^6^A methylation status of BCL2A1 was negatively correlated with IGF2BP2 gene expression. However, m^6^A methylation was not directly affected by the M^6^A-associated enzymes. Alternately, ALKBH5 is positively correlated with IGF2BP2. Collectively, based on our analysis, we propose that ALKBH5 regulates RBC differentiation and development by altering the methylation status of BCL2A1 via IGF2BP2. However, this requires further study and confirmation.

## Conclusion

M^6^A plays a significant role in thalassemia. In this study, we have, for the first time, explored the m^6^A RNA methylation status and its regulation of RNA stability in HbH-CS patients. Moreover, using m^6^A-RIP-seq and RNA-seq data, we established a profile of differentially methylated mRNA in T versus N samples. Lastly, we proposed involvement of m^6^A-regulated BCL2A1 in the pathogenesis of thalassemia. To confirm our preliminary data, more investigation is necessary to explore the methylation status of relevant RNAs and modulation of target downstream genes during erythroid differentiation.

Our work had certain deficiencies. Firstly, we discovered that there were two m^6^A methylation sites in BCL2A1. However, we did not elucidate which site holds more significance to thalassemia. Secondly, the underlying mechanism behind RNA methylation of BCL2A1 was not examined, and needs further exploration.

## Materials and methods

### Participants and samples

We collected samples from 16 HbH-CS thalassemia patients and 15 healthy volunteers, between March and July 2020. The HbH-CS patients had differing degrees of moderate anemia and hepatosplenomegaly and did not receive any blood transfusion in the past 3 months. The HGB range, among the HbH-CS patients, were between 56 and 103 g/l. In addition, Doppler's ultrasound of HbH-CS patients revealed splenomegaly ranging from Grade 1 to Grade 3. HbH-CS thalassemia and healthy volunteers will hereby be referred to as group T and group N, respectively. We conducted the Arraystar Human m^6^A-mRNA and lncRNA epitranscriptomic microarray analysis of 5 pairs of immature erythrocytes, particularly, 5 from HbH-CS thalassemia (T) and 5 from healthy volunteers (N). Our works received ethical approval from the First Affiliated Hospital of Guangxi Medical University. All participants were recruited from the same university and agreed to sign informed consent forms. If subjects were under 18, the informed consents were signed by their parent and/or legal guardian. All methods were performed in accordance with the Declaration of Helsinki. All HbH-CS patients were confirmed of their diagnosis with blood routine, haemoglobin electrophoresis, and DNA analysis^32,33^. All N groups were without any blood-related diseases. All participants were between the ages of 2 and 50. Patient demographics are provided in Table 1.

### Sorting of immature red blood cells (RBCs)

Immature RBCs were collected by sorting peripheral blood from the T and N cohorts, using a positive CD71 (CD71 Microbeads human, Miltenyi Biotec GmbH, Germany) selection method with a magnetic shelf (MiniMACS Starting Kit, Miltenyi Biotec GmbH, Germany), followed by confirmation with flow cytometry^34,35^. The flow cytometry results are depicted in Fig. 1. The sorted RBC samples were subsequently maintained in TRIZOL at − 80 °C for further analysis.

### Total RNA isolation and RT-qPCR

Total RNA was extracted with RNAiso Plus (Takara) and cDNA was synthesized with Prime Script TMRT reagent Kit with GDNA Eraser (Perfect Real-Time; Takara Bio, Shiga, Japan). Next, real time polymerase chain reaction (RT-qPCR) was conducted with the QuantiNova SYBR Green PCR Kit (QIAGEN, Product of Germany). Lastly, β-Actin was employed for normalization of gene expression.

### M^6^A Immunoprecipitation (MeRIP)

1–3 μg of total RNA and m^6^A spike-in control were introduced to 300 μL 1 × IP buffer (50 mM Tris–HCl, pH7.4, 150 mM NaCl, 0.1% NP40, 40U/μL RNase Inhibitor) with 2 μg of anti-m^6^A rabbit polyclonal Ab (Synaptic Systems). The solution was then maintained with head-over-tail rotation at 4 °C for 2 h. Meanwhile, 20μL (per sample) of Dynabeads™ with M-280 Sheep Anti-Rabbit IgG suspension was blocked with freshly made 0.5% BSA at 4 °C for 2 h, rinsed thrice with 300 μL 1 × IP buffer, and resuspended in the total RNA-antibody mixture described above. RNA was allowed to bind to the m^6^A-Ab beads during head-over-tail rotations at 4 °C for 2 h. Next, the beads were rinsed thrice with 500 μL 1 × IP buffer and twice with 500 μL wash buffer (50 mM Tris–HCl, pH7.4, 50 mM NaCl, 0.1% NP40, 40 U/μL RNase Inhibitor). Finally, the enriched RNA was eluted with 200 μL elution buffer (10 mM Tris–HCl, pH7.4, 1 mM EDTA, 0.05% SDS, 40U Proteinase K) at 50 °C for 1 h, before extraction and precipitation with acid phenol–chloroform and ethanol, respectively.

### Two-color RNA labeling and array hybridization

The modified RNAs were eluted from the immunoprecipitated magnetic beads as “IP” (immunoprecipitated, Cy5-labelled). The unmodified RNAs were recovered from the supernatant as “Sup” (supernatant, Cy3-labelled). The “IP” and “Sup” RNAs were then labeled with Cy5 and Cy3 respectively, in separate reactions, using Arraystar Super RNA Labeling Kit. The RNAs were combined together and hybridized onto Arraystar Human mRNA & lncRNA Epitranscriptomic Microarray (8 × 60 K, Arraystar). After washing the slides, the arrays were scanned in the two-color channels by an Agilent Scanner G2505C.

### Cell culture and transfection

K562 cell lines (Procell Biotechnology Co., Ltd, WUHAN, CHINA) were cultured as grown in RPMI medium 1640 basic (Gibco) supplemented with 10% fetal bovine serum (FBS) (Sijiqing, Zhejiang, China), and 1% penicillin–streptomycin (Solarbio, Beijing, China). Cells were transfected with siRNAs (final concentration: 20 nM) by riboFECT™ CP Reagent (RiboBio, GuangZhou, China) according to the manufacturer’s instructions. All siRNAs were obtained from Tsingke Biotechnology Co.Ltd. (nanning, China) shown below: IGF2BP2 sense: GAAGUGAUCGUCAGAAUUATT, antisense: UAAUUCUGACGAUCACUUCTT; siRNA Negative control (siCtrl), Sense: UUCUCCGAACGUGUCACGUTT, Antisense: ACGUGACACGUUCGGAGAATT.

IGF2BP2、ALKBH5 and BCL2A1 expression were confirmed by RT-Qpcr and Immunoblot.

### Immunoblotting

Cells were lysed using in RIPA buffer (Beyotime, Jiangsu, China). Proteins were electrophoretically resolved on 10% or 15% SDS–polyacrylamide gels (40 μg per lane), and transferred onto nitrocellulose membranes (Bio-Rad, Hercules, CA, USA). and electroblotted onto polyvinylidene difluoride (PVDF) membranes. After blocking with 5% BSA Blocking Buffer (Solarbio, Beijing, China)The membranes were washed 3 times with TBST (Solarbio, Beijing, China) with 1% Tween20. After incubation with appropriate first antibody 4 °C overnight and HRP-coupled secondary antibodies at room teperature for 1 h. Target proteins were detected in Gel and chemiluminescence dual imaging system (FluouChem HD2, Santa Clara, CA USA)and developed with BeyoECL Plus (Beyotime, Shanghai, China) and images analyzed with ImageJ (version 1.51j).

#### Reagents

Antibodies used for experiment were as follows: GAPDH (60004-1-1 g, 1:10,000)、 ALKBH5 (16837-AP, 1:2000), IGF2BP2 (11,601–1-AP, 1:2000) and HRP-coupled secondary antibodies (SA00001-1,1:2000) were from Proteintech Group, Inc (Wuhan, China). BCL2A1 (A0134,1:500) antibody was from Abclonal (Wuhan, China).

### Data analysis

The Agilent Feature Extraction software (version 11.0.1.1) was employed for the analysis of acquired array images. Raw intensities of Modified RNA (Cy5-labelled) and Unmodified RNA (Cy3-labelled) were normalized with the mean of log2-scaled Spike-in RNA intensities. Next, signals with Present (P) or Marginal (M) QC flags in a minimum of 1 in 10 samples were marked as “All Targets Value” in Excel for further “m^6^A methylation level”, “m^6^A quantity” and “expression level” calculations. The “m^6^A methylation level” was measured via the percentage of modification based on sample normalized intensities. The “m^6^A quantity” was assessed from the Modified RNA (Cy5-labelled) normalized intensities. The “RNA expression level” was analyzed according to the normalized intensities of all RNA. Moreover, we performed additional quartile normalization using the limma package to ascertain array expression, before probe flag screening. Differentially m^6^A-methylated RNAs or differentially expressed RNAs between the HbH-CS and healthy volunteers were recognized by fold change and statistical significance (p-value) values. Lastly, hierarchical clustering was done to show degrees of m^6^A-methylation within samples. P value < 0.05 denotes significance.

### Ethics approval and consent to participate

Our works received ethical approval from the First Affiliated Hospital of Guangxi Medical University. All participants agreed to sign informed consent forms.

### Consent for publication

All authors agree to the publication of this article.

## Supplementary Information

Supplementary Information 1.

Supplementary Information 2.

Supplementary Information 3.

Supplementary Information 4.

Supplementary Information 5.

Supplementary Information 6.

Supplementary Information 7.

Supplementary Information 8.

Supplementary Information 9.

## Data Availability

The following information was supplied regarding data availability. The sequencing data are available in the Supplemental Files.

## Abbreviations

HbH-CS: Hemoglobin H-constant spring disease
TDT: Transfusion dependent thalassemia
NTDT: Non-transfusiondependent thalassemia
BP: Biological process
CC: Cellular component
MF: Molecular function
IP: Immunoprecipitated
Sup: Supernatant
siCtrl: siRNA Negative control
