# Author Correction: Human m^6^A-mRNA and lncRNA epitranscriptomic microarray reveal function of RNA methylation in hemoglobin H-constant spring disease

**DOI:** 10.1038/s41598-021-01793-3

**Published:** 2021-11-10

**Authors:** Heyun Ruan, Fang Yang, Lingjie Deng, Dongmei Yang, Xiaoli Zhang, Xueyu Li, Lihong Pang

**Affiliations:** 1 Department of Obstetrics and Gynecology, The First Affiliated Hospital of Guangxi Medical University, No. 6 Shuangyong Road, Nanning, Guangxi China; 2 Department of Obstetrics and Gynecology, Minzu Hospital of Guangxi, Zhuang Autonomous Region, Affiliated Minzu Hospital of Guangxi Medical University, Nanning, Guangxi China

Correction to: *Scientific Reports* 10.1038/s41598-021-99867-9, published online 14 October 2021

The original version of this Article contained an error.

In the Materials and methods section, under subheading “Participants and samples”,

“Patient demographics are provided in Table 4.”

now reads:

“Patient demographics are provided in Table 1.”

The original Article has been corrected.

