# Supplementary figures and images for: Human m6A-mRNA and lncRNA epitranscriptomic microarray reveal function of RNA methylation in hemoglobin H-constant spring disease
Source: Sci Rep. 2021 Oct 14;11:20478. doi: 10.1038/s41598-021-99867-9 (PMC8516988; doi:10.1038/s41598-021-99867-9)

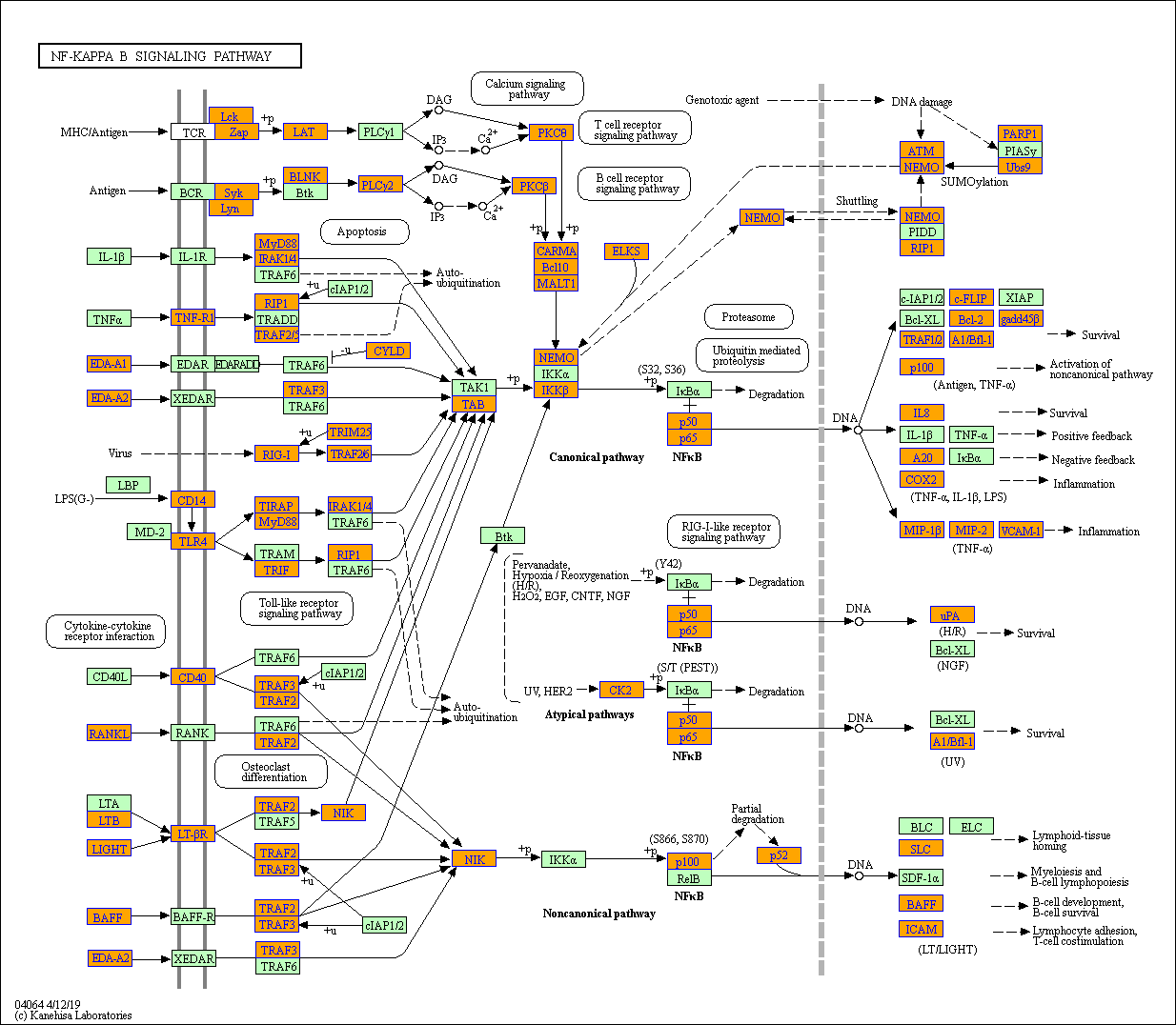

Supplement: Supplementary file 1 — Supplementary Information 1. [file 41598_2021_99867_MOESM1_ESM.png]

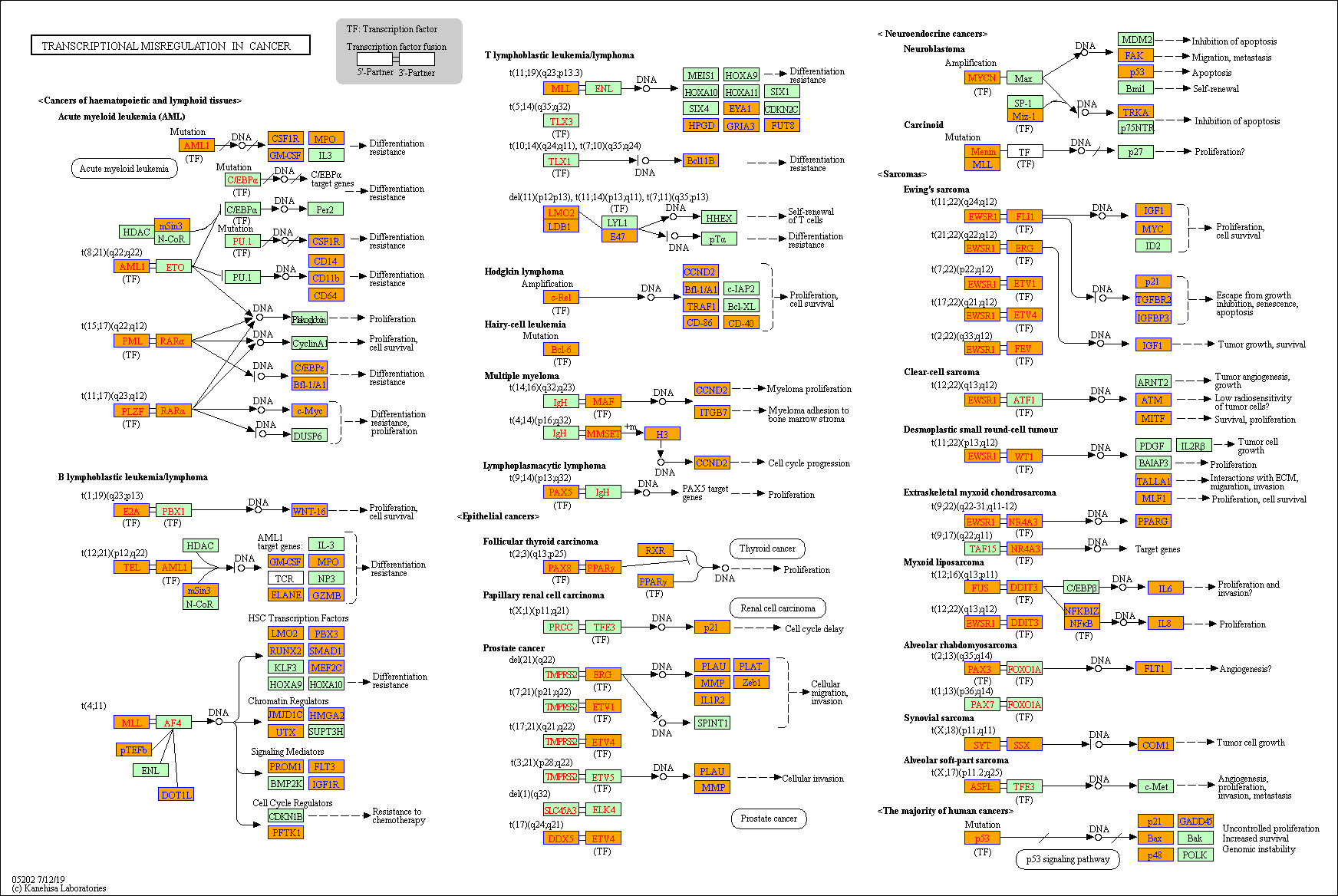

Supplement: Supplementary file 2 — Supplementary Information 2. [file 41598_2021_99867_MOESM2_ESM.png]

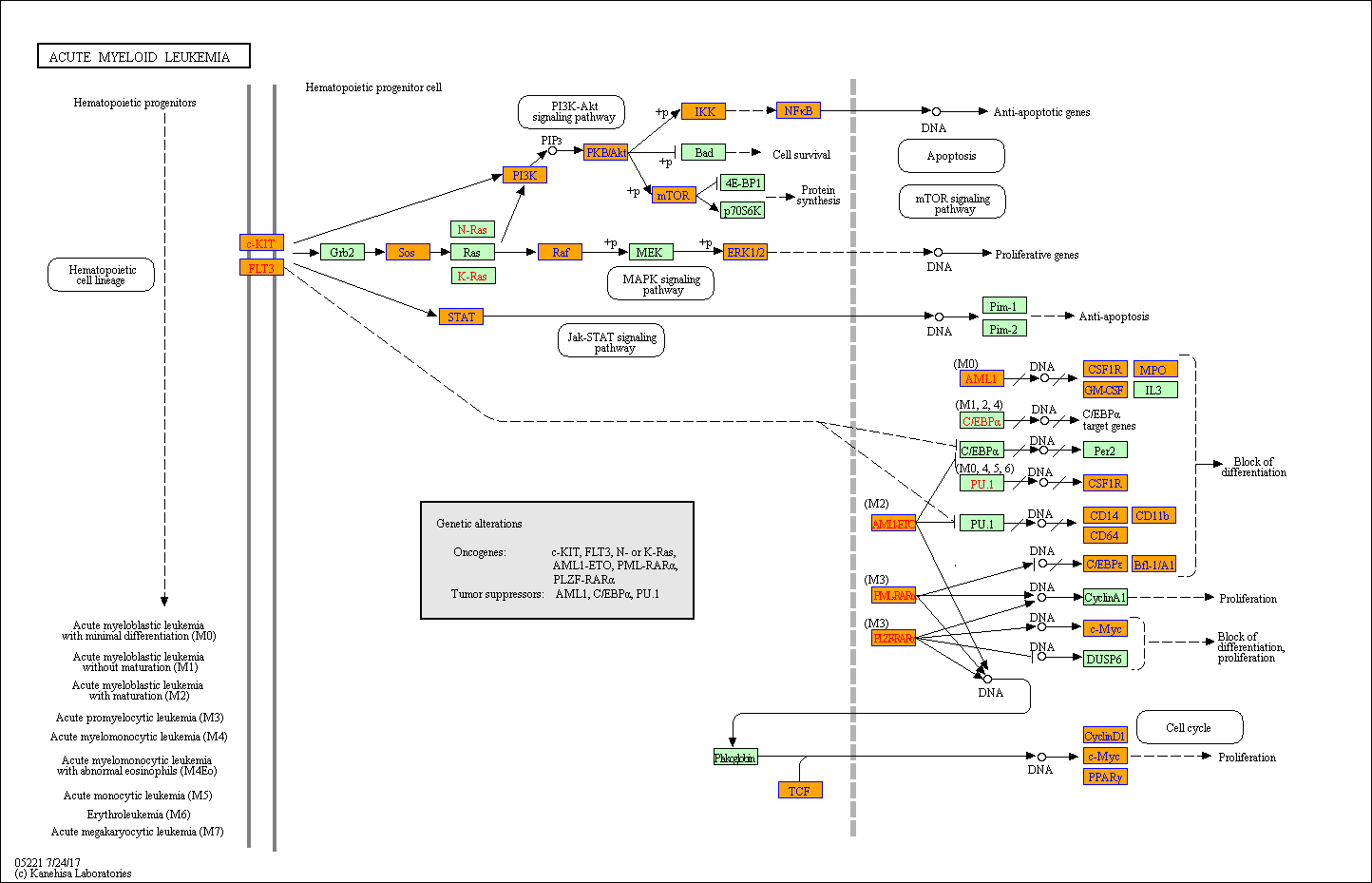

Supplement: Supplementary file 3 — Supplementary Information 3. [file 41598_2021_99867_MOESM3_ESM.png]

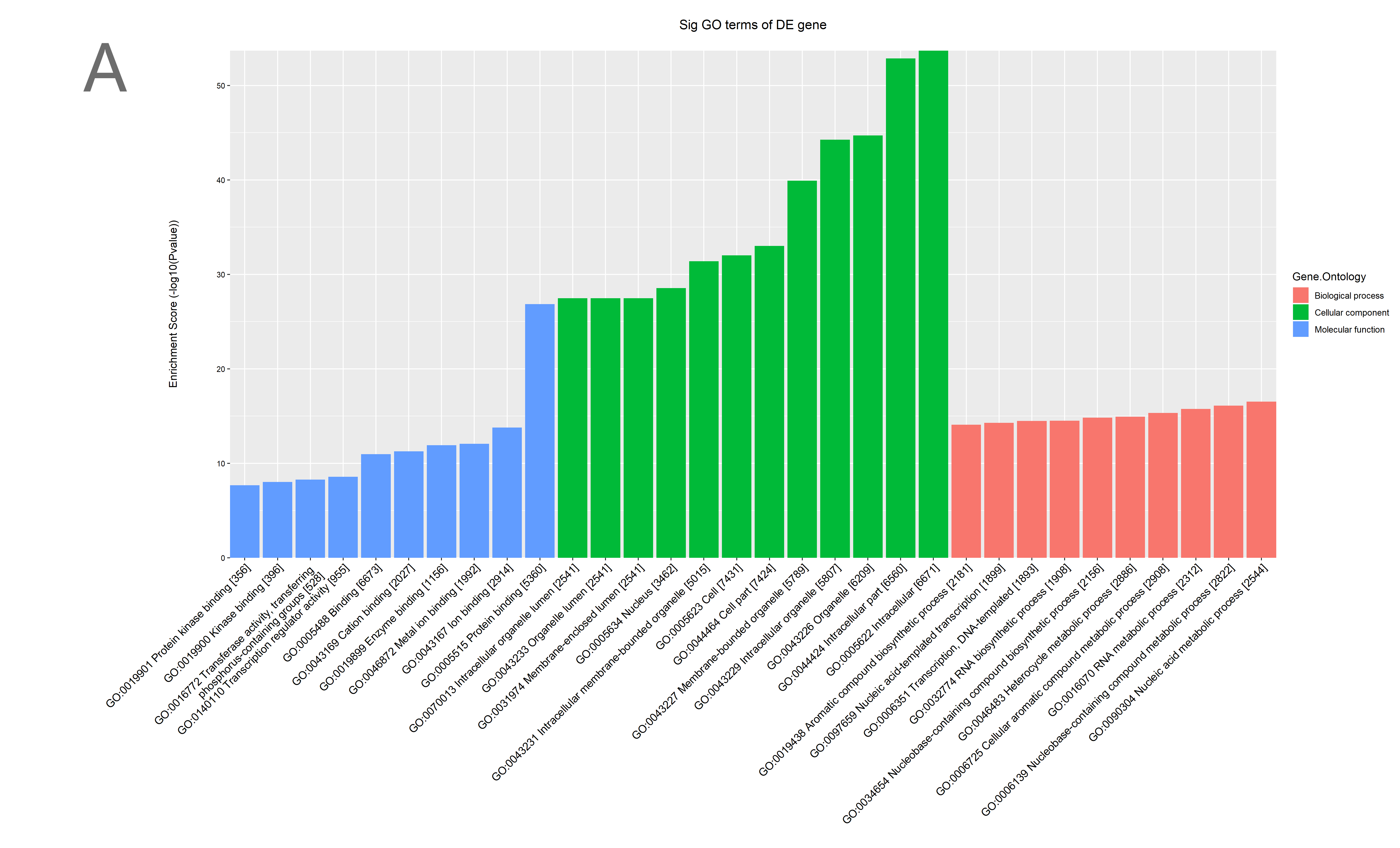

Supplement: Supplementary file 6 — Supplementary Information 6. [file 41598_2021_99867_MOESM6_ESM.png]

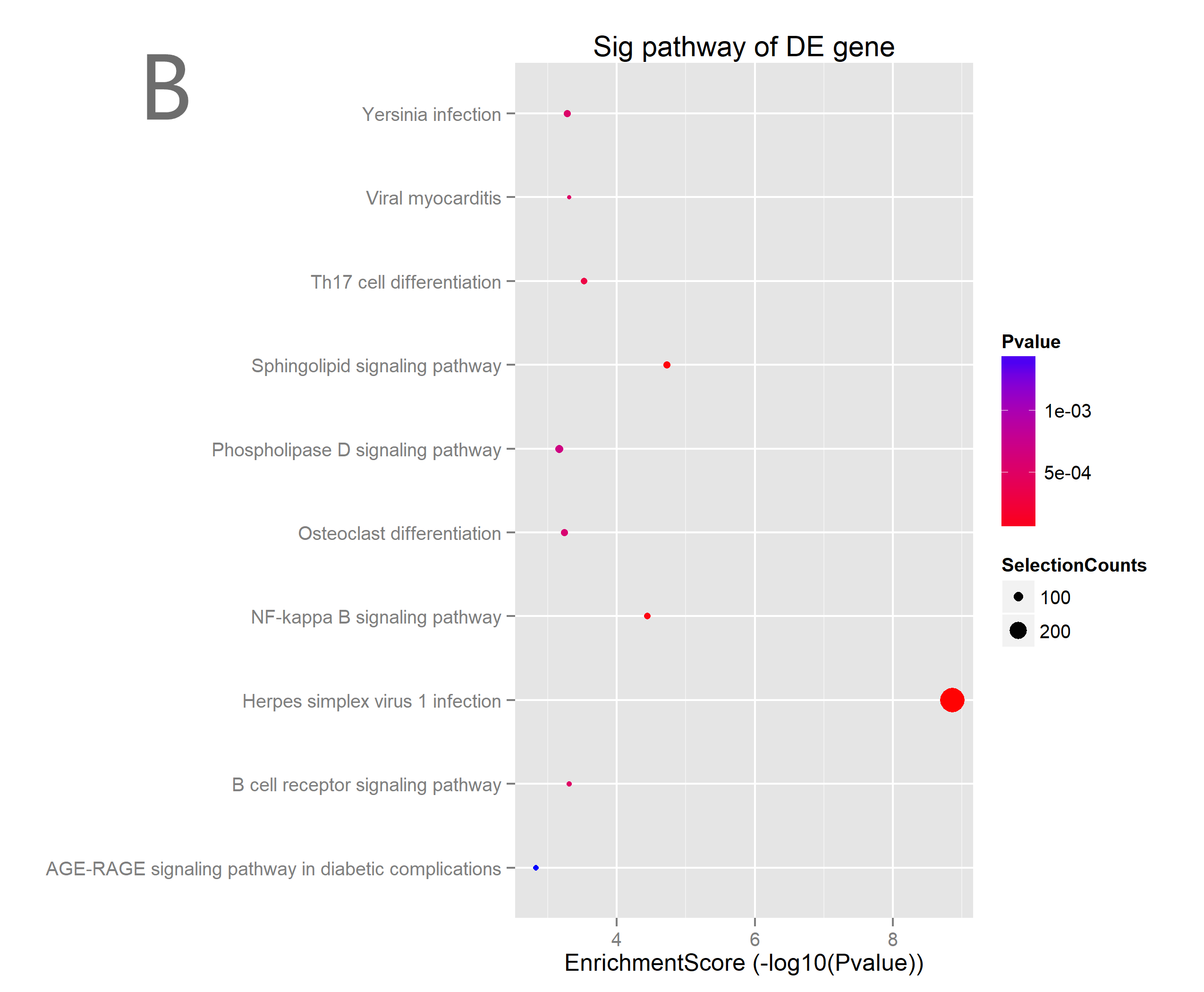

Supplement: Supplementary file 7 — Supplementary Information 7. [file 41598_2021_99867_MOESM7_ESM.png]

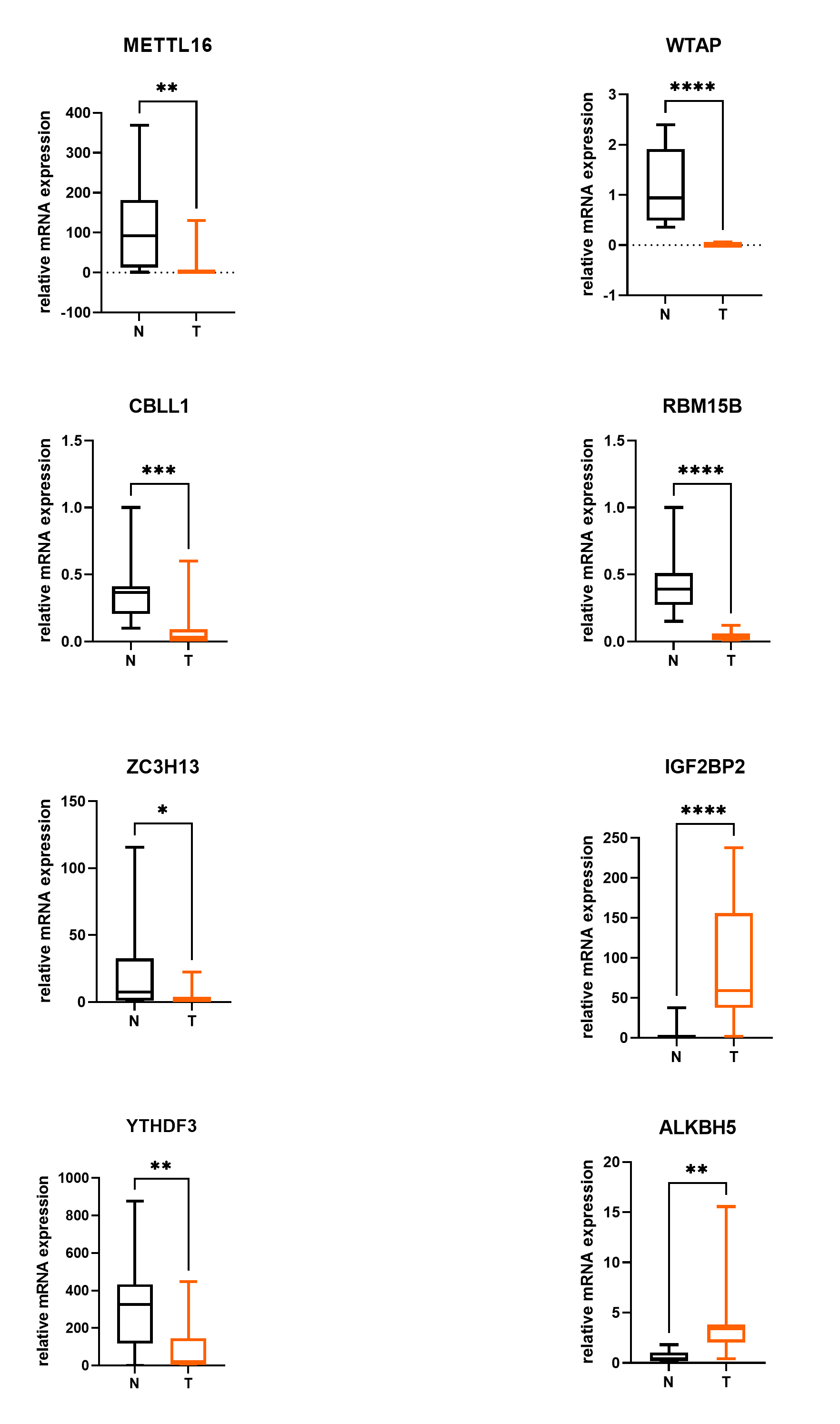

Supplement: Supplementary file 9 — Supplementary Information 9. [file 41598_2021_99867_MOESM9_ESM.tif]
