# Supplementary material for: Human m6A-mRNA and lncRNA epitranscriptomic microarray reveal function of RNA methylation in hemoglobin H-constant spring disease
Source: Sci Rep. 2021 Oct 14;11:20478. doi: 10.1038/s41598-021-99867-9 (PMC8516988; doi:10.1038/s41598-021-99867-9)
Supplement: Supplementary file 4 — Supplementary Information 4. [file 41598_2021_99867_MOESM4_ESM.pdf]

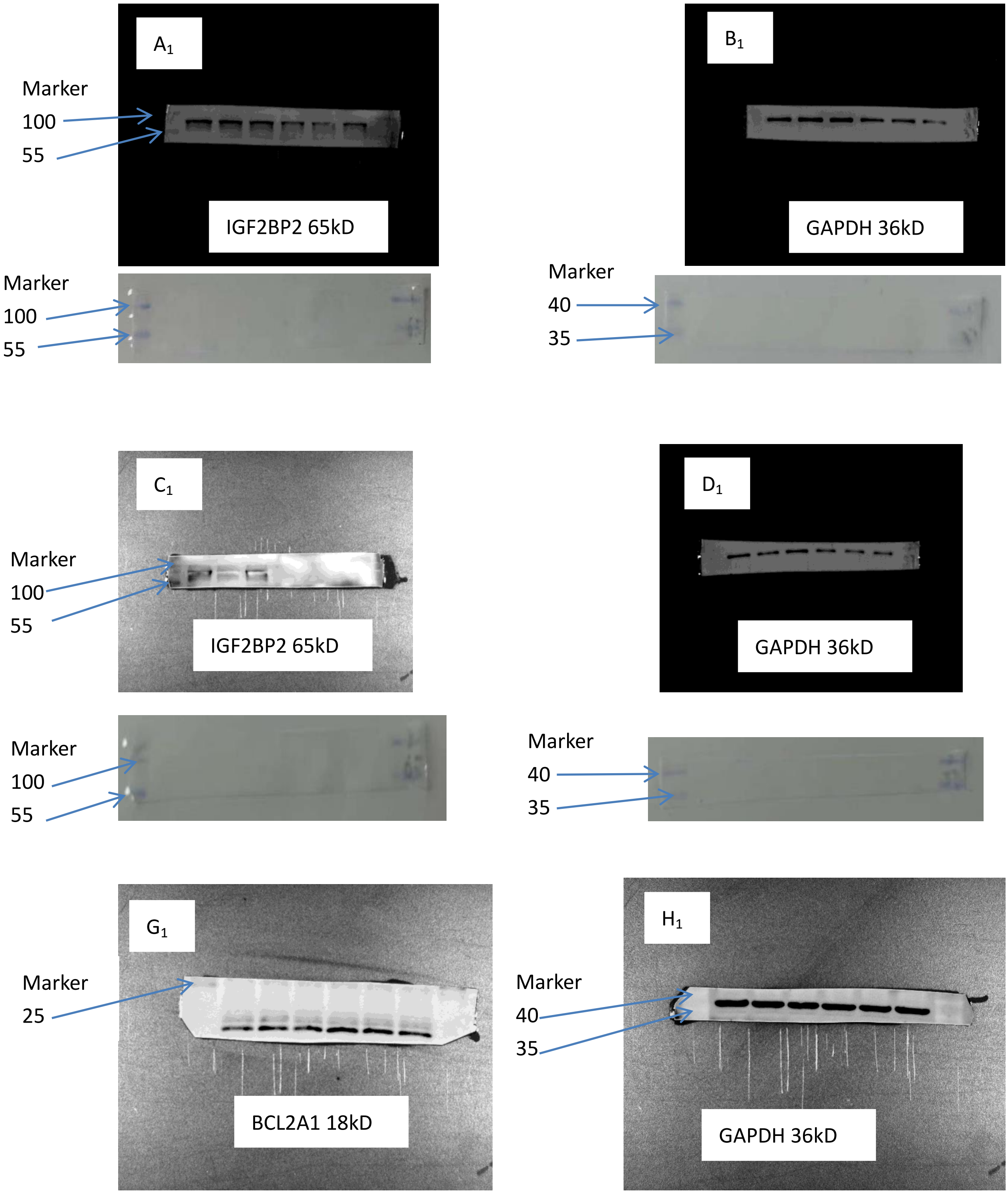
A1
Marker
100
55
IGF2BP2 65kD
Marker
100
55
B1
GAPDH 36kD
Marker
40
35
C1
Marker
100
55
IGF2BP2 65kD
Marker
100
55
D1
GAPDH 36kD
Marker
40
35
G1
Marker
25
BCL2A1 18kD
H1
Marker
40
35
GAPDH 36kD

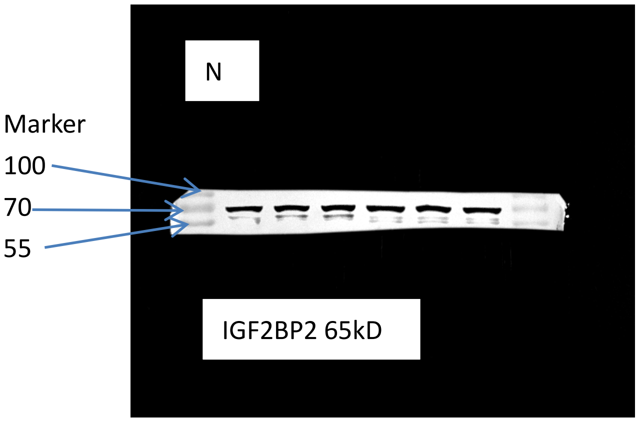

O

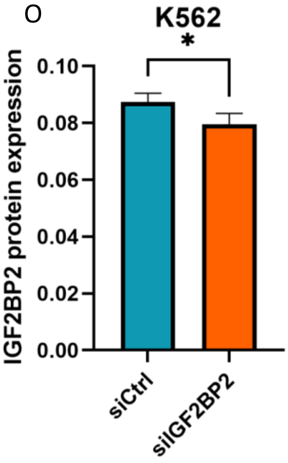

In order to explain the membrane can match the image, the additional image were shown by CLIN (ChemiScope6100Touch, Shanghai, China).Fig A1-D1were another image Corresponding to Fig A-D respectively. Fig G1、H1、N and O were from Fig M and Fig G1/H1 correspond to Fig G/H.
