# Supplementary material for: Human m6A-mRNA and lncRNA epitranscriptomic microarray reveal function of RNA methylation in hemoglobin H-constant spring disease
Source: Sci Rep. 2021 Oct 14;11:20478. doi: 10.1038/s41598-021-99867-9 (PMC8516988; doi:10.1038/s41598-021-99867-9)
Supplement: Supplementary file 5 — Supplementary Information 5. [file 41598_2021_99867_MOESM5_ESM.pdf]

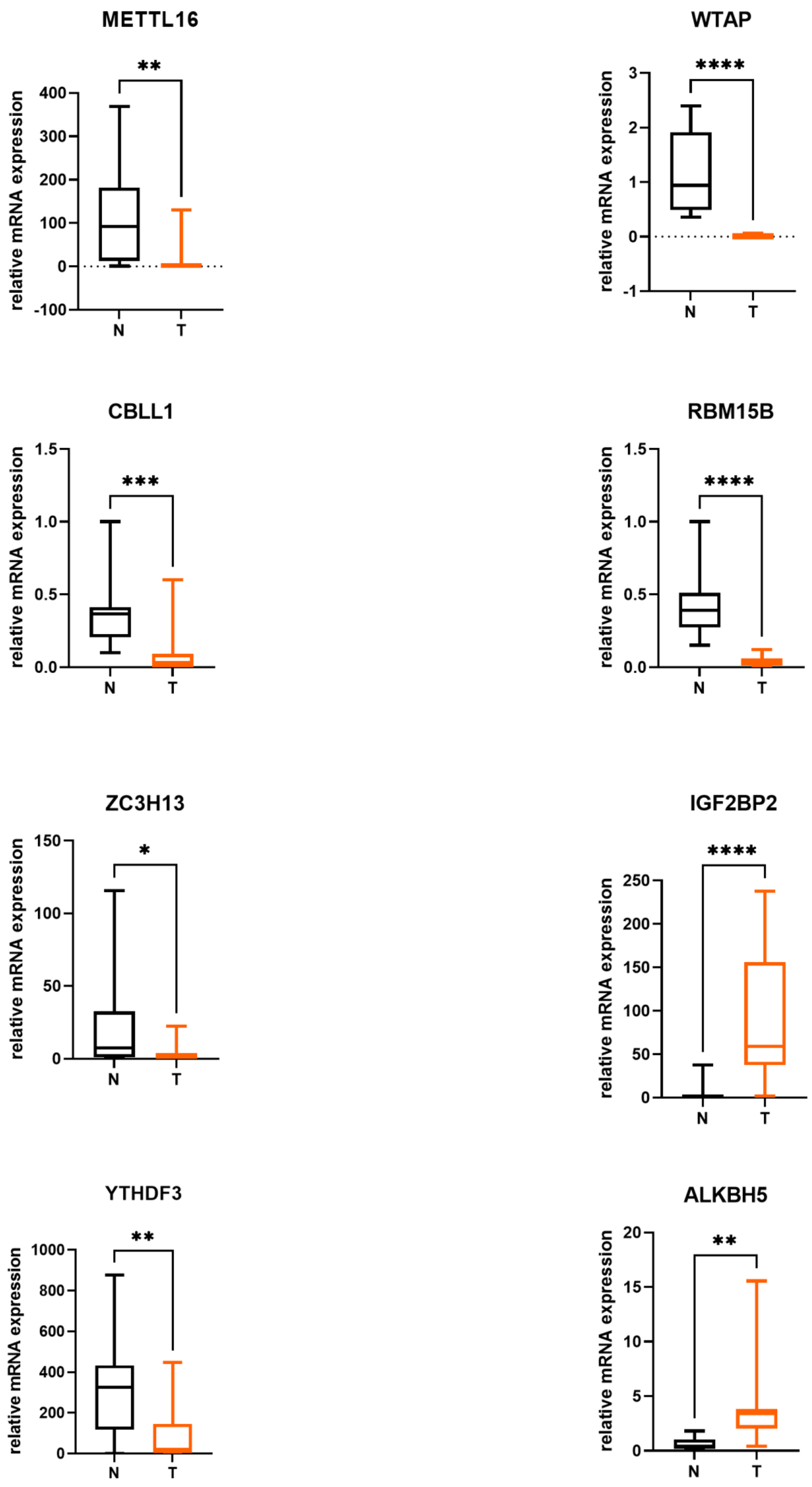

The differentially expressed profile of m6A-mRNAs in immature red blood cells of Hb CS thalassemia (T)and healthy volunteers controls (N) (**P*<0.05, ***P<0.01,* ****P*<0.01, *****P<0.0001*) . These genes were in Fig 3.
