# Supplementary material for: Human m6A-mRNA and lncRNA epitranscriptomic microarray reveal function of RNA methylation in hemoglobin H-constant spring disease
Source: Sci Rep. 2021 Oct 14;11:20478. doi: 10.1038/s41598-021-99867-9 (PMC8516988; doi:10.1038/s41598-021-99867-9)
Supplement: Supplementary file 8 — Supplementary Information 8. [file 41598_2021_99867_MOESM8_ESM.pdf]

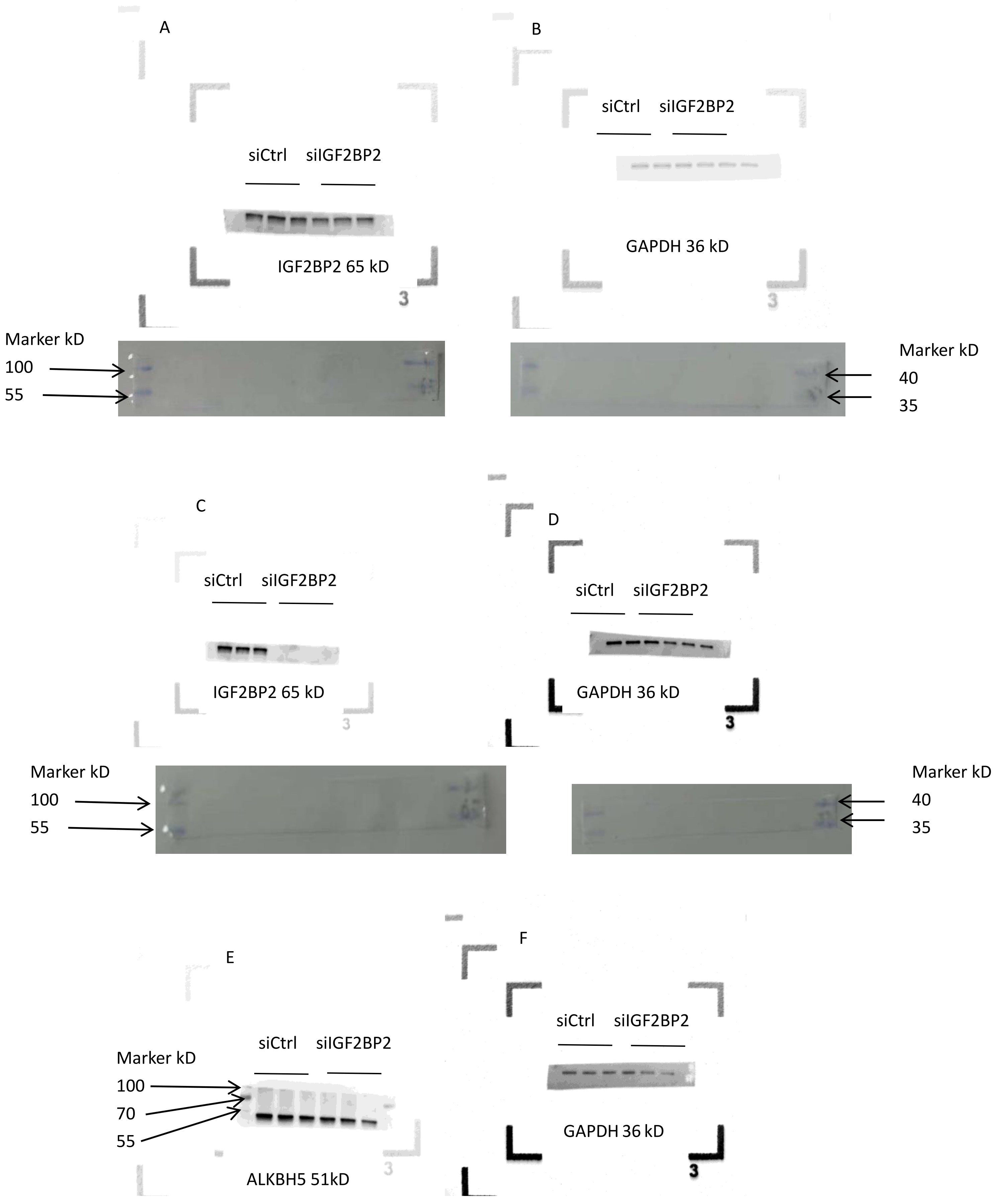
A
siCtrl siIGF2BP2
IGF2BP2 65 kD
Marker kD
100
55
B
siCtrl siIGF2BP2
GAPDH 36 kD
Marker kD
40
35
C
siCtrl siIGF2BP2
IGF2BP2 65 kD
Marker kD
100
55
D
siCtrl siIGF2BP2
GAPDH 36 kD
Marker kD
40
35
E
siCtrl siIGF2BP2
Marker kD
100
70
55
ALKBH5 51kD
F
siCtrl siIGF2BP2
GAPDH 36 kD

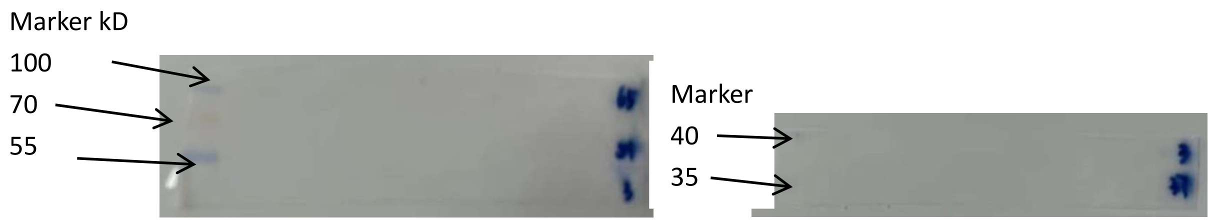

Marker kD
100
70
55
Marker
40
35

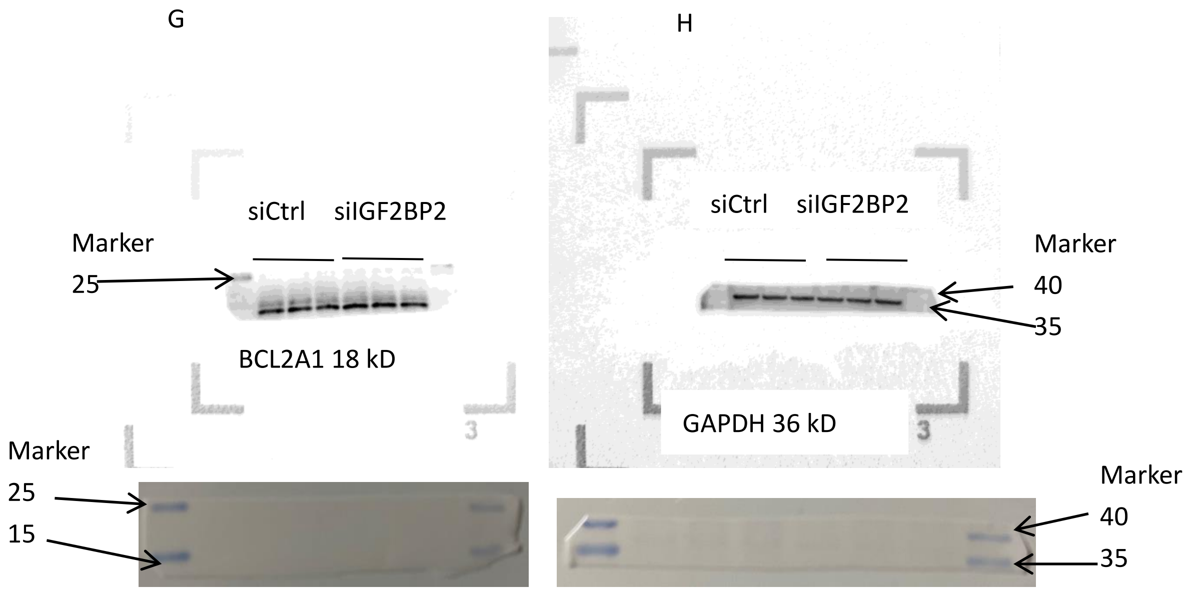

G
H
siCtrl
siIGF2BP2
Marker
25
BCL2A1 18 kD
siCtrl
siIGF2BP2
Marker
40
35
GAPDH 36 kD
Marker
25
15
Marker
40
35

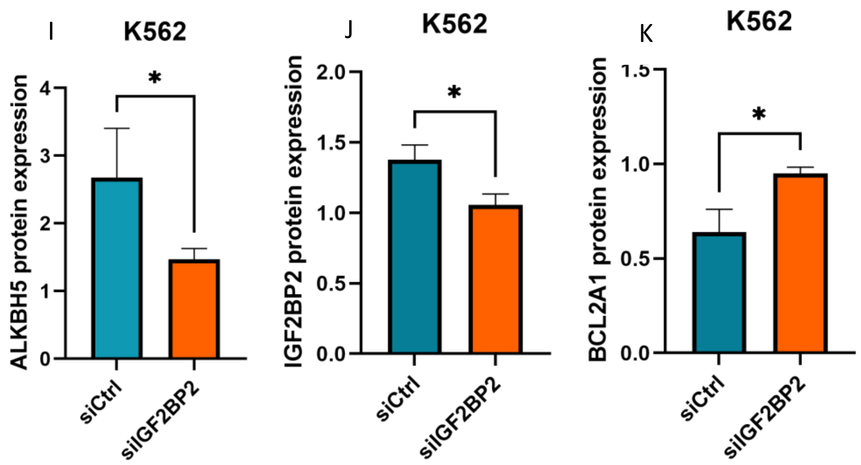

I
K562
ALKBH5 protein expression
*
siCtrl
siIGF2BP2
J
K562
IGF2BP2 protein expression
*
siCtrl
siIGF2BP2
K
K562
BCL2A1 protein expression
*
siCtrl
siIGF2BP2

L

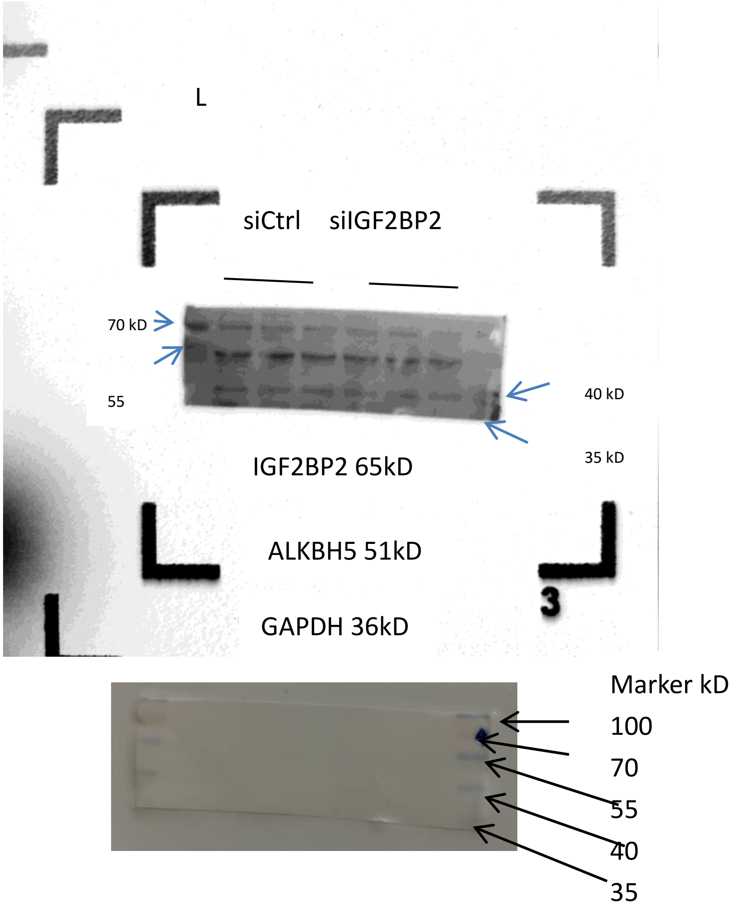

M

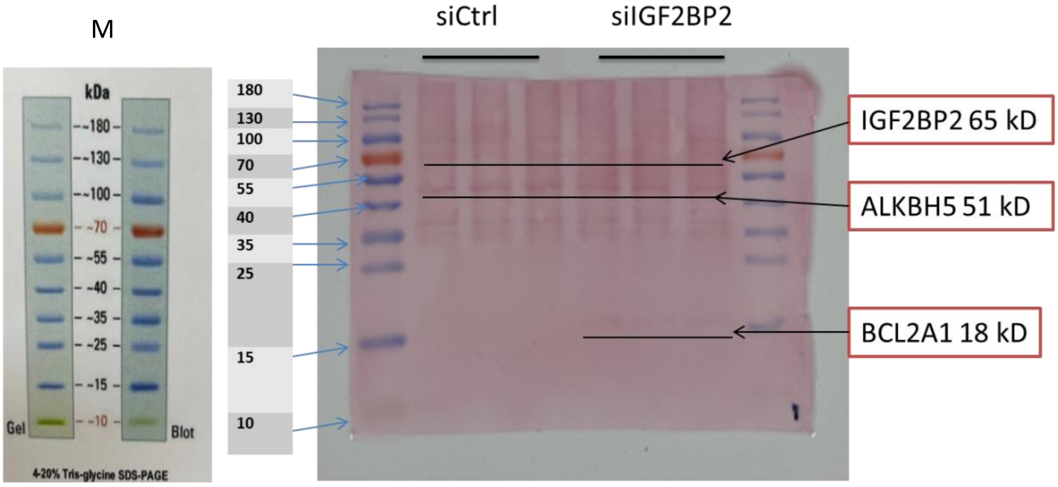

PageRuler Prestained Ladder 26616

15% Tris-glycine SDS-PAGE

Fig A and B were from the same membrane. Comparing the gray values of figure A and figure B, figure J can be obtained. Fig I came from fig E and F which were from the same membrane. Fig K came from fig G and H which were from the Fig M. Fig 5I IGF2BP2 and GAPDH figures were from

Fig C and D. ALKBH5 and GAPDH were from E and F. BCL2A1 and GAPDH were from G and H. The molecular weight of IGF2BP2、ALKBH5 and GAPDH were 65、51 and 36kD respectively. BCL2A1 can be observed 18 or 20kD. The original membranes were followed the image. The marker can be seen in the membranes, but can’t be observed in the image. Fig L was another membrane, some markers can be seen. They show three different molecular in the same membranes. Fig M showed some different protein in the whole membrane such as IGF2BP2、ALKBH5 and BCL2A1. In Fig M the membrane was stained by Ponceay Stain Reagent for 10 minute. The marker was 26616. Every strip was cut according the molecular in the appropriate range. The FluoChem HD2 was used to capture the image.
